# Effect of coenzyme Q10 supplementation on oxidative stress and clinical outcomes in patients with low levels of coenzyme Q10 admitted to the intensive care unit

**DOI:** 10.1017/jns.2021.39

**Published:** 2021-07-12

**Authors:** Mohammad Amin Valizade Hasanloei, Aidin Zeinaly, Mehran Rahimlou, Hadi Houshyar, Solma Moonesirad, Reza Hashemi

**Affiliations:** 1 Clinical Research Development Unit, Imam Khomeini Hospital, Urmia University of Medical Sciences, Ershad Ave, 5756151818 Urmia, West Azerbaijan Province, Iran; 2 Department of Anesthesiology, Urmia University of Medical Sciences, 11 km SERO Road, 5756151818 Urmia, West Azerbaijan Province, Iran; 3 Department of Nutrition, Faculty of Medicine, Zanjan University of Medical Sciences, Zanjan, Iran; 4 Department of Anesthesiology, Imam Khomeini Hospital, Faculty of Medicine, Urmia University of Medical Sciences, Imam Khomeini Avenue, 5756151818 Urmia, West Azerbaijan Province, Iran; 5 Urmia University of Medical Sciences, 11 km SERO Road, 5756151818 Urmia, West Azerbaijan Province, Iran

**Keywords:** Clinical trial, Coenzyme Q10, Mechanical ventilation, Trauma

## Abstract

Today, trauma is known to be the third leading cause of death in most countries. Studies have demonstrated below-normal plasma levels of antioxidants in trauma patients. The present study aimed to assess the efficacy of Coenzyme Q10 (CoQ10) on oxidative stress, clinical outcomes and anthropometrical parameters in traumatic mechanical ventilated patients admitted to the intensive care unit. Patients were randomised to receive sublingual CoQ10 (400 mg/d) or placebo for 7 d. Primary and secondary outcomes were measured at the baseline and end of the study. We enrolled forty patients for this trial: twenty in the CoQ10 group and twenty in the placebo group. There was not any significant difference in the baseline variables (*P* > 0⋅05). At the end of the study, CoQ10 administration caused a considerable reduction in the Malondialdehyde (MDA) and Interleukin 6 (IL-6) concentrations (*P* < 0⋅001), Glasgow Coma Score (GCS; *P* = 0⋅02), ICU and hospital length of stay and mechanical ventilation (MV) duration (*P* < 0⋅001). We found that CoQ10 administration could increase Fat-Free Mass (*P* < 0⋅001) (FFM; *P* = 0⋅04), Skeletal Muscle Mass (SMM; *P* = 0⋅04) and Body Cell Mass (BCM) percent (*P* = 0⋅03). There was not any significant difference in other factors between the two groups (*P* > 0⋅05). CoQ10 administration has beneficial effects on patients with traumatic injury and has no side effects. However, since the possibility of the type II error was high, the outcomes on the duration of MV, ICU stay and hospital stay, and GCS may very well be false positives.

## Introduction

Today, trauma is known to be the third leading cause of death in most countries, 50 % of which is due to brain damage^(1)^. Trauma exhibits a metabolic response that is characterised by hypermetabolism, inflammation^(2)^, oxidative stress^(3)^ and accelerated protein catabolism^(4)^. On the other hand, trauma is one of the crucial causes of admission in the intensive care unit. More than 30 % of the patients admitted to the intensive care unit have traumatic injuries^(5)^. Many of these patients require intubation and mechanical ventilation (MV). However, MV has a significant adverse effect on the physiological state of patients and may cause severe complications in patients, and therefore, the process of weaning from the ventilator in eligible patients should begin as soon as possible^(6,7)^. Long-term MV in patients with trauma causes exacerbation of oxidative stress and endothelial injury, which leads to mitochondrial dysfunction, tissue injury, organ failure and death^(8,9)^. Studies have demonstrated below-normal plasma levels of antioxidants in trauma patients and this cause prolonged ICU stay and increase mortality risk^(10,11)^.

Coenzyme Q10 (CoQ10), also known as ubiquinone, is an essential component of electron transfer in mitochondria that has antioxidant properties and its deficiencies interfere with mitochondrial function, which in turn leads to exacerbation of oxidative stress and cellular damage^(12)^. Also, CoQ10 deficiency in patients with septic shock is associated with controlled immune response and oxidative stress. Researchers have reported that CoQ10 prevents damage to cell membranes and improves vascular endothelial function^(13,14)^. Recently, the results of a cross-sectional study indicated a decrease in serum levels of CoQ10 in critically ill patients^(12)^.

To date, no randomised clinical trial (RCT) study has been conducted to evaluate the effect of CoQ10 supplementation in traumatic mechanical ventilated patients and we hypothesised that CoQ10 administration in these patients could have beneficial effects on biochemical and clinical factors. So, we investigated the effects of CoQ10 supplementation on plasma levels of Malondialdehyde (MDA), Interleukin 6 (IL-6), clinical outcomes and anthropometrical parameters in traumatic mechanical ventilated patients admitted to the intensive care unit.

## Methods

### Participants and study design

**Fig. 1.**
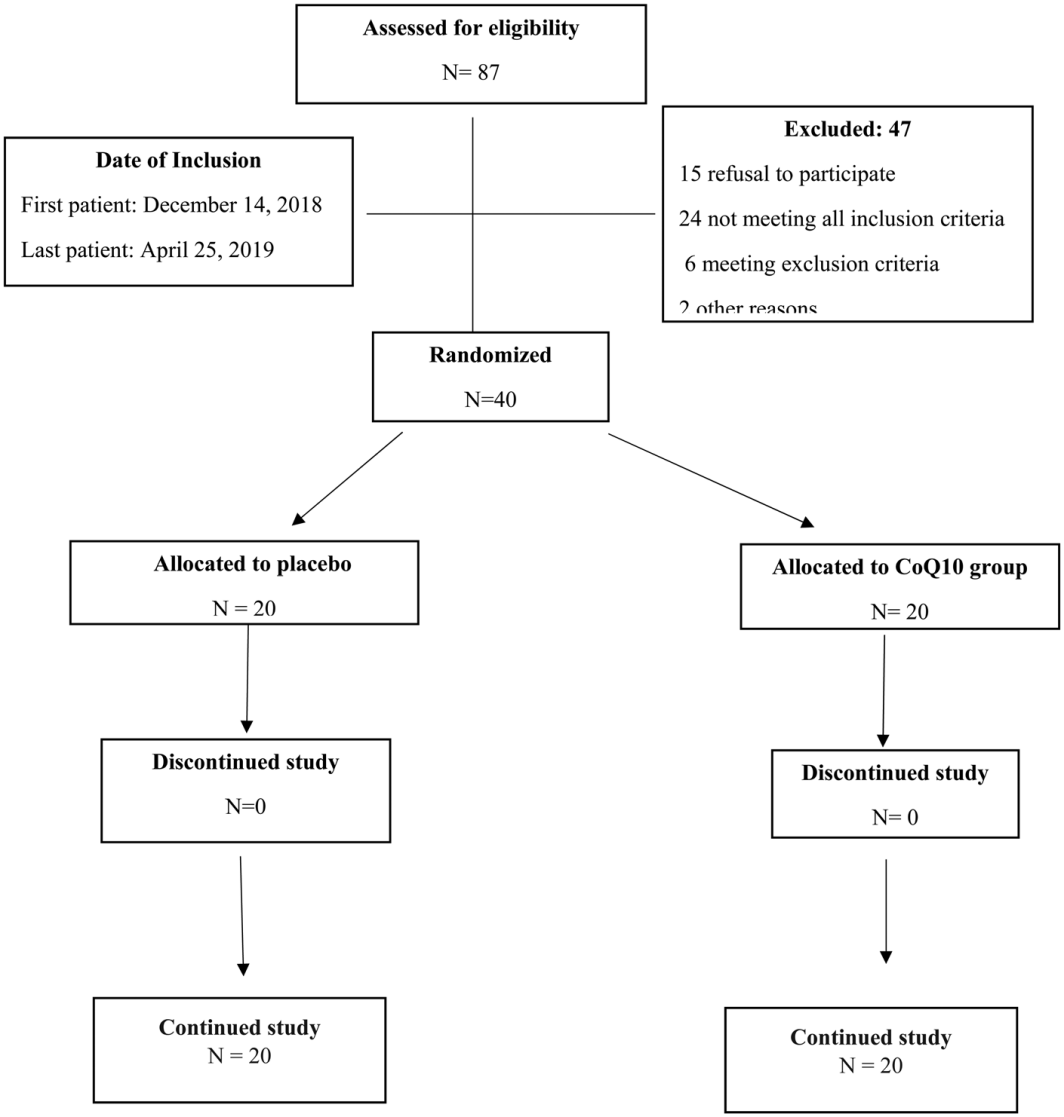
Summary of patient flow diagram.

The present study was conducted according to the guidelines laid down in the Declaration of Helsinki and all procedures involving human subjects/patients were approved by the Urmia University of Medical Sciences Ethics Committee and Clinics Institutional Review Board approved the trial (ir.umsu.rec.1397.134) and registered in the Iranian Registry of Clinical Trials (IRCT20171221037983N4 and registration URL: https://www.irct.ir/trial/38173). Informed consent was obtained from all patients or their family. From September 2019 to January 2020, forty traumatic injury patients with sub-therapeutic plasma CoQ10 levels (defined as <2⋅5 μg/ml) from Imam Khomeini Hospital Complex were enrolled in this trial. Fig. 1 shows the flow diagram of the study. The inclusion criterion was adult patients (age: 18–65 years) with an expected need of MV for at least 48 h and at least 7 d stay in the ICU and GCS equal or greater than 7. The exclusion criteria were a previous history of acute or chronic kidney diseases, haemodialysis, pneumonia, GCS <7, taking statins or anticoagulant and immunosuppressive drugs, pregnancy, taking omega 3, CoQ10 or other antioxidant supplements and contraindication for enteral feeding.

Based on the Mean (± sd) of IL-6 for intervention and placebo groups in a previous study^(15)^ (2⋅83 ± 1⋅34 pg/ml in the placebo group and 1⋅67 ± 0⋅75 pg/ml in the intervention group) with type I error (*α*) of 5 % and a power of 90 %, the sample size was calculated twenty subjects in each group using the following formula:

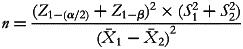


The forty enrolled patients underwent block randomisation, using a computer-generated sequence: two were allocated in the intervention group and twenty in the placebo group. The patients in the intervention group were received 400 mg/d CoQ10 (Capsule 100 mg, Product of Nutri Century, Canada, every 6 h by the sublingual route) and a control group that received placebo for 7 d. The dose was chosen based on safety data, showing that doses of 400 mg are well-tolerated in patients^(16)^. Given the lack of human trials in patients with traumatic injury, we did not use a very high dose of CoQ10. The safety of doses of 600, 800 and 1200 mg/d CoQ10 has been shown in other diseases, with the most significant benefit in the group receiving the highest dosage (1200 mg/d)^(17,18)^. The allocation of the patients to the CoQ10 or placebo group was concealed to the patients, researchers, nurses of the intensive care unit and laboratory staff. Both CoQ10 and placebo capsules were identical in appearance. All of the included patients had sub-therapeutic plasma CoQ10 levels, a mean of 1⋅3 μg/ml and range 0⋅8–2⋅2 μg/ml.

The energy requirement of all patients was calculated as 25 kcal/kg. Enteral feeding for patients in two groups began in the first 24 h of admission and the early 72 h of admission; nutritional requirements were administered as 50 % carbohydrate, 30 % fat and 20 % protein. The patients received all routine care under the direction of their admitting physicians throughout the study.

### Patient and public involvement

Patients were not involved in the design, conduct or interpretation of the study.

### Assessment of outcomes

The concentration of IL-6 was considered as the primary outcome. The mean of the Sequential Organ Failure Assessment (SOFA) and GCS score, MV duration, ICU length of stay, hospital length of stay and body composition and concentration of other biochemical biomarkers were recognised as the secondary outcomes.

### Biochemical assessment

Fasting venous blood samples (10 ml) were obtained from all of the patients at the beginning and end of the study. Serum and plasma were prepared after centrifugation (3000 rpm, 4 °C, 15 min) and were then stored at −80 °C until analysis. CoQ10 levels were measured in plasma samples by the Urmia University of Medical Sciences Biochemistry Research Center (Urmia, Iran) using high-performance liquid chromatography as previously described in detail^(19)^.

Serum levels of MDA were determined by the Thiobarbituric acid reactive substances (TBARS) assay using tetraethoxypropane as standard^(20)^. Also, for evaluation of the IL-6 concentration, we used the ELISA kits (Diaclone Research, Besançon, France).

### Anthropometric measurements

Patients’ weight was measured using the Seca 984 bed scales, and body composition was measured by the InbodyS10 body analyzer. Body composition-related markers include FFM, Arm Circumference, SMM, PBF and BCM.

### Clinical measures

SOFA^(21)^ and GCS were evaluated using a scoring system at the beginning and end of the study. Also, MV duration and the mortality rate were evaluated at the end of the study.

### Statistical analysis

Quantitative variables were reported as mean values with their standard deviations or standard error, and qualitative variables were presented as frequency (%). Normality was checked by Kolmogorov–Smirnov and Shapiro–Wilk tests. The *χ*^2^ test was used for the comparison of the categorical variables between three groups, and the Fischer test was used to compare the mortality rate between the two groups. The within-group comparison was analysed using the paired *t* test or Wilcoxon test. Pre-treatment and post-treatment variables were compared between groups using the Independent *t* test or Mann–Whitney *U* test. Statistical significance was set at *P* < 0⋅05, based on two-sided tests. The analysis was carried out with SPSS 20⋅0.

## Results

### Characteristics of the participants

From December 2018 to April 2019, eighty-seven adult traumatic patients were screened from Imam Khomeini Hospital Complex ICU; forty-seven were not enrolled (for some reasons such as plasma CoQ10 levels >2⋅5 μg/ml, unwillingness of the patient or family to participate in the study and having exclusion criteria) and finally, the inclusion criteria of the present study were met by forty participants. No patients were lost to follow up in the present trial (Fig. 1). No side effects were reported following administration of CoQ10 and placebo capsules. The baseline demographic and clinical characteristics of the subjects are shown in Table 1. There was no significant difference in the baseline demographic and clinical characteristics between the two groups (*P* > 0⋅05). The subjects recruited were aged between 22 and 63 years, with a mean (±sd) age of 52⋅47 ± 7⋅26 years in the intervention group and 50⋅12 ± 9⋅66 years in the control group that was not significantly different (*P* = 0⋅28). Sixty-five percent of participants in the control group and 50 % in the intervention group were men (*P* = 0⋅34). Also, there were no significant differences in the subjects’ baseline GCS and SOFA score (*P* = 0⋅86 and *P* = 0⋅07, respectively).

**Table 1.** Comparison of the baseline and demographic characteristics between two groups

Variable	Intervention (*n* 20)	Control (*n* 20)	*P*-value _trend_
Gender (Male, *n* (%))	10 (50 %)	13 (65 %)	0⋅34*
Age (year)	52⋅47 ± 7⋅26^¥^	50⋅12 ± 9⋅66	0⋅28^¶^
GCS	7⋅75 ± 0⋅91	7⋅8 ± 0⋅77	0⋅86^¶^
SOFA	7⋅3 ± 1⋅08	6⋅55 ± 1⋅36	0⋅07^¶^
Weight (kg)	72⋅4 ± 11⋅68	72⋅9 ± 11⋅22	0⋅89^¶^
Diagnosis, *n* (%)			
Multiple trauma (no TBI)	16 (80)	15(75)	0⋅76*
Trauma including TBI	4 (20)	5 (25)	0⋅67*
Body Composition			
FFM (%)	50⋅54 ± 9⋅57	50⋅1 ± 10⋅65	0⋅93^¶^
Arm circumference (cm)	29⋅1 ± 4⋅88	29⋅32 ± 4⋅11	0⋅9^¶^
SMM (%)	26⋅68 ± 5⋅56	25⋅84 ± 6⋅19	0⋅71^¶¶^
PBF (%)	29⋅77 ± 10⋅26	31⋅02 ± 10⋅72	0⋅64^¶¶^
BCM (%)	30⋅79 ± 5⋅91	32⋅36 ± 6⋅13	0⋅43
Clinical Biomarkers			
IL-6 (pg/ml)	175⋅05 ± 40⋅15	177⋅82 ± 30⋅05	0⋅73
MDA (pg/ml)	232⋅37 ± 70⋅36	239⋅96 ± 67⋅37	0⋅79

BCM, Body Cell Mass; FFM, Free Fat Mass; GCS, Glasgow Coma Scale; IL-6, Interleukin 6; MDA, Malondialdehyde; PBF, Percent Body Fat; SOFA, Sequential Organ Failure Assessment score; SMM, Skeletal Muscle Mass; TBI, Traumatic Brain Injury.

¥ Values are presented as Mean (± sd).

* *P*-values for Pearson's *χ*^2^ test.

¶ *P*-values for Independent *t* test.

¶¶ *P*-values for Independent samples Mann–Whitney *U* test.

### CoQ10 levels

As shown in Table 2, the baseline CoQ10 levels in the two groups were similar. At the end of the study, CoQ10 administration compared with the control group caused a significant increase in plasma total CoQ10 concentration (*P* < 0⋅001).

**Table 2.** Comparison of the baseline and after intervention characteristics within two groups

Variable	Intervention group (*n* 20)			Placebo group (*n* 20)			*P*-value^3^
	Before	After	*P*-value^1^	Before	After	*P*-value^2^	
SOFA	7⋅3 ± 1⋅08	4⋅25 ± 1⋅62	<0⋅001	6⋅55 ± 1⋅36	5⋅25 ± 1⋅21	<0⋅001	<0⋅001
GCS	7⋅75 ± 0⋅91	11⋅15 ± 1⋅84	0⋅01	7⋅8 ± 0⋅77	9⋅6 ± 1⋅54	0⋅04	0⋅02
Weight (kg)	72⋅4 ± 11⋅68	72⋅62 ± 11⋅72	0⋅39	72⋅9 ± 11⋅22	71⋅55 ± 10⋅67	0⋅15	0⋅23
Body Composition							
FFM (%)	50⋅54 ± 9⋅57	57⋅25 ± 8⋅46	<0⋅001	50⋅1 ± 10⋅65	47⋅25 ± 10⋅41	0⋅04	<0⋅001
Arm circumference (cm)	29⋅20 ± 3⋅77	28⋅23 ± 3⋅69	0⋅43	28⋅93 ± 3⋅77	26⋅88 ± 2⋅84	0⋅13	0⋅69
SMM (%)	26⋅68 ± 5⋅56	30⋅53 ± 4⋅76	0⋅03	25⋅84 ± 6⋅19	23⋅74 ± 5⋅74	0⋅05	0⋅04
PBF (%)	29⋅77 ± 10⋅26	25⋅45 ± 8⋅84	<0⋅001	31⋅02 ± 10⋅72	25⋅67 ± 10⋅78	<0⋅001	0⋅61
BCM (%)	30⋅79 ± 5⋅91	35⋅58 ± 4⋅47	0⋅01	32⋅36 ± 6⋅13	30⋅53 ± 6⋅53	0⋅08	0⋅003
Clinical Variables							
IL-6	175⋅05 ± 40⋅15	98⋅6 ± 33⋅44	<0⋅001	177⋅82 ± 30⋅05	160⋅47 ± 30⋅22	0⋅79	<0⋅001
MDA	232⋅37 ± 70⋅36	143⋅53 ± 18⋅74	<0⋅001	239⋅96 ± 67⋅37	213⋅73 ± 58⋅7	0⋅035	0⋅001

BCM, Body Cell Mass; BMR, Basal Metabolic Rate; FFM, Free Fat Mass; GCS, Glasgow Coma Scale; PBF: Percent Body Fat; SOFA, Sequential Organ Failure Assessment score; SMM, Skeletal Muscle Mass.

¥ Values are presented as Mean (± sd).

1,2 *P*-value, difference compared with the value at the beginning of the study within groups (paired *t* test).

3 *P*-value, mean difference of changes between the two groups (independent *t* test).

### Effect of CoQ10 supplementation on MDA and IL-6 concentrations

The baseline mean (±sd) plasma IL-6 concentration was 175⋅05 ± 40⋅15 pg/ml in the CoQ10 and 177⋅82 ± 30⋅05 pg/ml in the control group, and there was not any significant difference (*P* = 0⋅73). As shown in Table 2, after the 7 d of intervention, the improvement in the IL-6 concentration in the CoQ10 group was significantly higher than compared with the control group (−76⋅99 ± 8⋅81 pg/ml *v.* −17⋅35 ± 3⋅33 pg/ml, *P* < 0⋅001).

At the beginning of the study, there was no significant difference between two groups in the MDA concentration (232⋅37 ± 70⋅36 pg/ml in the CoQ10 group and 239⋅96 ± 67⋅37 pg/ml, *P* = 0⋅79). At the end of the intervention, comparisons showed that serum values of MDA reduced were significantly higher in the CoQ10 group than compared with the control group (−88⋅84 ± 14⋅9 pg/ml *v.* −26⋅23 ± 7⋅04 pg/ml, *P* = 0⋅001).

### Effect of CoQ10 supplementation on clinical measures

While the power analysis was not designed to reliably determine the effect of CoQ on clinical outcome variables, and thus a type II error is certainly possible, we nevertheless report the following results.

As shown in Table 2, after the 7 d of intervention, CoQ10 supplementation significantly improved the SOFA score (−3⋅5 ± 1⋅32 *v.* −1⋅30 ± 0⋅66, *P* = 0⋅04). Also, at the end of the study, both groups had a significant increase in the GCS score; however, this increase was significantly higher in the CoQ10 group (3⋅4 ± 1⋅6 *v*. 1⋅8 ± 1⋅24, *P* = 0⋅02).

The results of the present study were showed that CoQ10 supplementation compared with the control group caused a significant reduction in the MV duration (6⋅85 ± 2⋅58 *v.* 9⋅8 ± 4⋅05, *P* < 0⋅001). Also, the CoQ10 group compared with the control group showed a significant lower ICU length of stay (9⋅4 ± 3⋅26 d *v.* 13⋅35 ± 5⋅55 d, *P* < 0⋅001) and hospital length of stay (13⋅2 ± 7⋅65 d *v.* 19⋅15 ± 8⋅46 d, *P* < 0⋅001; Table 3).

**Table 3.** Comparison of the average ICU length of stay and intubation duration (days) between two groups

Variable	Intervention group (*n* 20)	Placebo group (*n* 20)	*P*-values
ICU length of stay (days)	9⋅4 ± 3⋅26	13⋅35 ± 5⋅55	<0⋅001^¶^
Hospital length of stay (days)	13⋅2 ± 7⋅65	19⋅15 ± 8⋅46	0⋅02^¶^
Days of mechanical ventilation (days)	6⋅85 ± 2⋅58	9⋅8 ± 4⋅05	<0⋅001^¶¶^

¶ Independent *t* test.

¶¶ Mann–Whitney *U* test.

### Effect of CoQ10 supplementation on the body composition

Body composition parameters are presented in Table 2. CoQ10 supplementation compared with the control group significantly improved FFM (6⋅72 ± 1⋅44 % *v.* −2⋅86 ± 0⋅65 %, *P* < 0⋅001), SMM (4⋅04 ± 0⋅94 % *v.* −2⋅1 ± 0⋅38 %, *P* = 0⋅04) and BCM (4⋅79 ± 0⋅97 % *v.* –1⋅66 ± 0⋅49 %, *P* = 0⋅03) percent. However, there was not any significant difference between the two groups in weight (*P* = 0⋅23), arm circumference (*P* = 0⋅69) and PBF (*P* = 0⋅61).

## Discussion

We investigated the effect of CoQ10 supplementation with a dose of 400 mg/d for 7 d on clinical and body composition parameters in traumatic mechanical ventilated patients admitted to the intensive care unit. To the best of our knowledge, this is the first RCT reporting the improvement in the MDA and IL-6 concentrations, GCS and SOFA scores, and MV duration, and FFM, SMM and BCM percent following administration of CoQ10.

Previous studies have shown that the levels of inflammatory factors increase in patients with traumatic injury^(22,23)^. Researchers have suggested that elevated blood cytokine concentrations during the acute phase of trauma are correlated with the development of fatal post-traumatic complications, especially MV duration^(24,25)^. On the other hand, critical illness, such as trauma with or respiratory distress, significantly increases the production of reactive oxygen species (ROS) and weakens the body's antioxidant system^(26)^. The results of the present study showed that supplementation with CoQ10 compared with placebo significantly reduced the levels of IL-6 and MDA, as the main indicators of inflammatory and oxidative stress responses. We evaluated the concentration of MDA by TBARS assay. TBARS is probably the oldest and one of the most widely used assays for measuring the MDA level. However, this method is subject to interferences. In human plasma, sialic acid present in glycoprotein can interfere, but such interference can be minimised by carrying out the reaction in a phosphoric acid medium. On the other hand, some other confounding factors, especially oxidised lipids, saturated and unsaturated aldehydes, sucrose, and urea impair the accuracy of the results evaluated with this method. For this reason, the use of chromatographic separation of MDA–thiobarbituric acid adduct method is suggested that we used this method^(27,28)^. In a cross-sectional study in 2012, Lee *et al.* evaluated the association between plasma coenzyme Q10 levels and oxidative stress-related factors in patients with coronary heart disease. The results of their study showed that CoQ10 was inversely correlated with the levels of oxidative stress-related factors, especially MDA^(29)^. Studies carried out in clinical settings among patients with heart failure, and other diseases show a beneficial effect of CoQ10 supplementation on inflammatory and oxidative stress factors. Bor-Jen Lee *et al.* have shown that 150 mg/d CoQ10 supplementation for 12 weeks in patients with coronary artery disease caused a significant reduction in the MDA concentration and higher catalase and superoxide dismutase activity^(30)^. A clinical trial study in patients with Type 2 Diabetes (T2DM) showed that COQ10 supplementation (100 mg/d, 12 weeks) had a significant effect on 8-isoprostane and MDA concentrations^(31)^. CoQ10 is a potent antioxidant that can reduce oxidative injury by free radicals and strengthens the body's antioxidant system^(32)^. Also, Donnino *et al.* have shown that the provision of 200 mg enteral ubiquinol (the reduced form of CoQ10) for up to 7 d in patients with severe sepsis or septic shock had a beneficial effect on inflammatory biomarkers^(33)^. Previous studies have suggested that the anti-inflammatory effects of CoQ10 are due to its inhibitory effect on NF-κB gene expression^(34)^, attenuating miR-146a and IL-1 receptor-associated kinase modulation^(35)^ and inhibition of the protein-1 alpha releasing from the macrophage^(36)^.

Our finding showed that CoQ10 supplementation compared with placebo was reduced MV duration significantly. In relation to clinical variables, due to the low sample size in each group and since the possibility of the type II error was high, the outcomes on the duration of MV, ICU stay and hospital stay, and GCS may very well be false positives.

Pulmonary dysfunction in trauma patients is multifactorial, and oxidative stress and inflammation can worsen this process^(7)^. On the other hand, hypoxia itself can exacerbate inflammation and damage the organs^(37)^. MV is a life-saving technique provided to critically ill patients to obtain a sufficient exchange of pulmonary gas and to relieve excessive respiratory muscle activity^(38–40)^. However, various studies have shown that prolonged MV in critically ill patients can damage the lung tissue and cause a disorder so-called ventilator-induced lung injury (VILI). Recently, a similar concern has emerged about the potential adverse effects of invasive MV on the respiratory system is the occurrence of ventilator-induced diaphragmatic dysfunction (VIDD)^(41–43)^. It has been shown that MV alone reduces diaphragm strength and contributes to prolonging MV even in the absence of trauma/sepsis^(44,45)^. The mechanism of this type of ‘intrinsic’ diaphragm dysfunction is also thought to be at least in part related to oxidative stress and mitochondrial dysfunction^(46,47)^, so if CoQ10 is indeed having an impact on the duration of MV/ICU stay/Hospital stay, it may be due to an effect it is having on this ‘intrinsic’ ventilator-induced diaphragm dysfunction (VIDD)^(48)^.

CoQ10 plays an essential role in the electron transport chain as the carrier of electrons from complex I and II to complex III in the inner mitochondrial membrane. Decreased CoQ10 levels in critically ill patients can impair mitochondrial function and lead to decreased levels of cellular adenosine triphosphate (ATP) production^(13)^. Farazi *et al.* in a clinical trial have demonstrated that CoQ10 administration (200 mg/d for 14 d) in critical patients with Community-Acquired Pneumonia (CAP) led to an improvement in clinical biomarkers. They showed that at the end of the study, the absolute numbers of patients who needed MV was low in the CoQ10 group^(48)^.

In this trial, CoQ10 administration caused a significant reduction in the length of ICU stay and the length of hospital stay. Contrary to the results of our study, Farazi *et al.* reported that 200 mg/d CoQ10 administration in elderly patients with pneumonia could not decrease the length of hospital stay^(48)^. Also, Donnino *et al.* not found any significant difference in the length of ICU stay and the length of hospital stay after the 200 mg enteral ubiquinol administration in patients with severe sepsis or septic shock^(33)^. Probably, one reason for the discrepancy between the results of our study with previous findings is the difference in the CoQ10 dose used in our study. However, several studies have reported a direct association between decreased plasma CoQ10 levels and an increased risk of mortality in critically ill patients^(49–51)^. Various theories have been reported regarding the favourable effect of CoQ10 in reducing the mortality rate. Most patients admitted to the ICU and Cardiac Care Unit (CCU) use statin family medications. Long-term use of these drugs can impair mitochondrial function^(52,53)^. Statins change lipid metabolism through impairing HMG-CoA reductase, the rate-limiting enzyme essential for cholesterol synthesis in the mevalonate pathway. This pathway also generates isoprenylated protein and CoQ10 proteins. The concentration of blood CoQ10 decreased throughout statin therapy. A review of 8 placebo-controlled CoQ10 studies have found a mean reduction of −0⋅44 μmol/l (95 % CI −0⋅52, −0⋅37 μmol/l)^(54)^. Also, long-term use of statins in critically ill patients causes muscle weakness and symptoms known as Statin-Associated Muscle Symptoms (SAMS)^(55)^. Administration of CoQ10 can prevent side effects of statins, especially myopathy and mitochondrial dysfunction^(56)^. CoQ10 improves mitochondrial function by enhancing the expression of sirtuin1, peroxisome proliferator-activated receptor-γ coactivator 1α and sirtuin3 genes that all of which prevent the progression of age-related diseases^(57)^.

Body composition is altered during critical illness. These changes include loss of muscle mass, reduction in the body weight, changes in body water and body cell mass^(58)^. We found that CoQ10 administration could increase FFM, SMM and BCM percent significantly. The results of our evaluations showed that no previous studies had examined the efficacy of CoQ10 supplementation on body composition in critically ill patients. However, some studies have been performed on the elderly population and athletes. Ravaglia *et al.* examined the association between serum CoQ10 level and body composition-related variables in the elderly. The results of the study showed that the decrease in plasma CoQ10 concentration was associated with a decrease in FFM percent^(59)^. CoQ10 in mitochondria is involved in carbohydrate, fat and protein metabolism. Some studies have shown that increased mitochondrial concentration of coenzyme CoQ10 improves body composition-related parameters^(60,61)^.

In the present study, for the first time, the effect of high-dose CoQ10 supplementation in patients with traumatic injury was investigated. We also evaluated the plasma concentration of CoQ10 at the beginning and the end of the study and did not include patients with higher plasma levels of CoQ10 at the beginning of the study to eliminate the confounding effect. However, the present study has some potential limitations.

With only twenty patients per group, the chance of a type II error (i.e. a false positive finding) for clinical outcomes variables is quite high. Since the possibility of a type II error is high, the outcomes on the duration of MV, ICU stay and hospital stay, and GCS may be false positives. Also, due to the lack of a similar study, the sample size was calculated based on a previous study^(15)^ performed on cancer patients rather than traumatic patients. Moreover, it was also better to evaluate the changes in antioxidant enzymes activity. Despite the thiobarbituric acid (TBA) test for MDA determination being the most frequently used method to evaluate lipid peroxidation, it shows several pitfalls and has been criticised as being too unspecific and prone to artifacts. TBA can react with several compounds, including sugars, amino acids, bilirubin and albumin, producing interferences in the measurement^(62)^. Finally, the duration of therapy was relatively short (maximum of 7 d), and a longer duration and follow-up period may be needed.

## Conclusions

We have shown that CoQ10 could improve some of the clinical and anthropometric parameters in patients with a traumatic injury. It also caused a significant reduction in ICU and hospital length of stay and MV duration. Further investigation with different dosages and longer intervention time may be warranted to determine the efficacy of CoQ10 in patients with a traumatic injury.
